# Benefits and limitations of humanized mice in HIV persistence studies

**DOI:** 10.1186/s12977-020-00516-2

**Published:** 2020-04-06

**Authors:** Matthew D. Marsden

**Affiliations:** Department of Microbiology and Molecular Genetics and Department of Medicine (Division of Infectious Diseases), School of Medicine, University of California, Irvine, CA USA

**Keywords:** HIV, Latency, Humanized mice, In vivo, Models, Persistence, Cure, BLT

## Abstract

Significant advances in the treatment of HIV infection have been made in the last three decades. Antiretroviral therapy (ART) is now potent enough to prevent virus replication and stop disease progression. However, ART alone does not cure the infection, primarily because HIV can persist in stable long-term reservoir cells including latently-infected CD4 + T cells. A central goal of the HIV research field is to devise strategies to eliminate these reservoirs and thereby develop a cure for HIV. This requires robust in vivo model systems to facilitate both the further characterization of persistent HIV reservoirs and evaluation of methods for eliminating latent virus. Humanized mice have proven to be versatile experimental models for studying many basic aspects of HIV biology. These models consist of immunodeficient mice transplanted with human cells or tissues, which allows development of a human immune system that supports robust infection with HIV. There are many potential applications for new generations of humanized mouse models in investigating HIV reservoirs and latency, but these models also involve caveats that are important to consider in experimental design and interpretation. This review briefly discusses some of the key strengths and limitations of humanized mouse models in HIV persistence studies.

## Introduction

Human immunodeficiency virus (HIV) infection depletes the CD4 + T cells that serve as hosts for the virus, eventually leading to the development of acquired immunodeficiency syndrome [1]. Potent antiretroviral therapy (ART) consisting of combinations of antiretroviral drugs that are taken daily is now available, which suppresses HIV replication and prevents disease progression [2]. However, replication-competent HIV can persist in rare latently-infected CD4 + T cells and potentially other reservoirs over years of ART [3–5], meaning that ART alone does not cure HIV infection. Issues including the development of virologic drug resistance, high financial cost, side-effects of ART drugs, elevated levels of immune activation despite therapy, adherence/treatment fatigue, and continued social stigma can all affect people living with HIV and taking daily ART. Moreover, many HIV-infected people around the world do not have access to these life-saving therapies [2]. Developing an HIV cure is therefore a major focus for the field.

## HIV latency and persistence

Latently-infected cells harbor integrated copies of HIV provirus that typically express little or no RNA and no viral proteins, but are capable of producing infectious virions if the cell is stimulated [4–6]. These cells also decay very slowly during ART and can maintain the infection for decades [7], possibly proliferating through clonal expansion via homeostatic or antigen-driven mechanisms during this time [8]. Other potential reservoirs capable of maintaining HIV over long periods of time during ART are less-well defined but might include some level of ongoing virus replication in tissues such as lymph nodes [9] or anatomic reservoirs such as the central nervous system [10], where the primary infected cell types are perivascular macrophages and microglial cells.

A wide range of experimental strategies are being investigated to achieve the challenging goal of curing HIV. These include gene therapy approaches intended to disrupt HIV proviral sequences [11] or critical host genes required for infection with many HIV strains such as CCR5 [12], transplantation approaches to replace the immune system with HIV-resistant cells [13], genetically enhancing the antiviral immune response [14], “kick and kill” [15] approaches to induce expression of latent virus and then kill the host cell, and “block and lock” [16] strategies to permanently silence residual proviral genomes (reviewed in [17, 18]). These approaches are initially developed and tested using in vitro tissue culture systems. However, such in vitro models do not provide the complex tissue architecture, cell–cell interactions, metabolism, compartmentalization, circulation, and diverse cell and tissue types present in vivo. Therefore in vivo testing is critical for evaluating the efficacy of promising strategies identified through in vitro studies.

Given our incomplete understanding of HIV persistence during therapy and the pressing need to develop methods to eliminate viral reservoirs that remain during ART, tractable in vivo models are required. One popular approach to this problem is to utilize humanized mice. The goal of this article is to provide a brief and general overview of the major benefits and limitations of modern, commonly used humanized mouse models in HIV persistence research.

## Benefits of humanized mice

Currently used model systems to study HIV in vivo include non-human primates and humanized mice. Humanized mice consist of immunodeficient mice that are transplanted with human cells or tissues, allowing them to be infected with HIV. Many different types of humanized mice exist, which differ in the specifics of the background mouse strain, transplanted human cell or tissue type, timing of transplant, and procedures used in humanization (reviewed in [19, 20]). However, all are intended to recapitulate key aspects of the human immune system. These models have advanced significantly since the early pioneering studies [21–24] that originally established this experimental approach and applied it to study HIV. Use of humanized mouse models has enhanced our understanding of HIV gene function, replication, cellular tropism, pathogenesis, and treatment, and are increasingly being applied to studies directed towards HIV persistence and cure [19]. As is the case for most scientific models, humanized mice have a variety of benefits and limitations that should be carefully considered when designing experiments and interpreting results (Table 1).

**Table 1:** Broad benefits and limitations of humanized mouse models in HIV persistence research

Benefits	Limitations
***In vivo model***	***Small animal size limits sample volumes and cell numbers that can be obtained***
– Recapitulates complexities not possible with in vitro systems	– Bleeds during experiment are restricted to several hundred microliter volumes
– Allows experimental interventions and sampling not feasible in clinical studies	– Rare cell subsets such as latently-infected resting CD4+ T cells may require pooling of tissues for analysis in some tissues
***Supports robust infection with wild-type HIV***	***Nature of xenograft may interfere with some experimental approaches***
– Virus used for infection does not require genetic modification to overcome host species barriers	– Most tissues outside of the reconstituted human immune system are murine
– Routes of infection are the same as in humans (allowing transmission or reservoir establishment studies)	– Graft-versus-host disease may develop and restrict timescale of experiments (time to GVHD is dependent on host strain and experimental model)
– Clinically relevant antiretroviral drug regimens can be directly tested	
– Reagents such as unmodified HIV-specific broadly neutralizing antibodies can be evaluated	
***Contains human HIV target cell types***	***Limited lifespan of mice***
– All major HIV host cell types are present, including CD4+ T cells and macrophages	– Depending on model, experiments lasting over 1 year are often infeasible
– Infection causes CD4+ T cell depletion and other HIV-related immune defects, allowing mechanisms of HIV persistence and pathogenesis to be investigated	– HIV reservoir changes occurring only over long periods of time would likely not be captured in these models
***Forms latent HIV reservoirs in CD4+ T cells***	***Mice are individually humanized by transplantation of human cells and/or tissues***
– Allows testing of latency reversing agents	– Most advanced models require surgical techniques
– Viral rebound occurs if ART is stopped	– Requires sources of human hematopoietic stem cells
***Reconstituted with human immune cytotoxic cells***	***Restrictions on the use of fetal tissues***
– Efforts to augment HIV-infected cell killing through natural killer cells or CD8+ T cells can be evaluated	– Models requiring implantation of human fetal tissues or cells are subject to substantial restrictions in some parts of the world
***Allows testing of human-specific gene or cytokine therapy approaches***	***Some immune cell lineage reconstitution and immune responses are incomplete***
– Gene therapy approaches specific for human genes can be directly evaluated (tailoring sequences to animal models for preclinical evaluation is not required)	– While newer models are improving this deficit, adaptive immune responses including IgG production and cytotoxic T cell responses have historically been difficult to elicit
– Human specific cytokines can be evaluated for effects on human cells in vivo without species-related receptor-ligand incompatibilities	– Reconstitution with macrophage and natural killer cells is limited
***Permits large “N” to discern differences in experimental groups***	***Murine metabolism differs from human***
– >30 animals can be constructed in a single series from the same human donor cells/tissue	– Pharmacokinetic characteristics and drug metabolism in mice and human are different
– Small animal size allows small amounts of experimental drug and compound to be used in pilot studies versus larger animal models	

Humanized mice are powerful tools for studying HIV persistence and latency. However, as for all model systems they are more suitable for addressing some questions than others as summarized here

The major motivator for using humanized mice to study HIV is that they provide an in vivo model which permits experimental interventions and sampling that are not feasible in clinical studies of infected people. The viral strain, dose, route of infection, sampling times, experimental interventions (such as ART administration or anti-latency therapies), and cells/tissues to be analyzed can all be defined with precision. Tissue samples from any organ are available for analysis, including those that can typically not be obtained in clinical studies. This is particularly important since only around 2% of the CD4 + T cells in the body are circulating in the peripheral blood, and most HIV infection and replication occurs in tissues, which are also the predominant location of HIV reservoir cells [25].

Advanced humanized mouse models such as bone marrow-liver-thymus (BLT) mice [26] are reconstituted with an extensive complement of human immune cells in many tissues throughout the mouse. This means that the interaction between wild-type HIV and human host cells including CD4 + T cells and macrophages can be studied in vivo and do not require prior genetic modification to overcome virus species barriers. Therefore clinically relevant antiretroviral drugs, broadly neutralizing antibodies, and other reagents that are specific to HIV can be directly tested, without needing to modify the reagent or virus as sometimes is required for evaluation in simian immunodeficiency virus (SIV) or simian-human immunodeficiency (SHIV) model systems [27].

Infection of humanized mice can be achieved through various methods including the intravenous, intravaginal, or intrarectal routes that are most relevant to human HIV transmission. These mice can therefore be used in transmission studies focused for example on topical antiretroviral drugs or pre-exposure prophylaxis [28–30] and potentially for evaluations of HIV reservoir establishment soon after primary infection through physiologically relevant routes.

The main cell types that are infected by HIV in vivo are CD4 + T cells and macrophage-lineage cells [31, 32]. These human cells are present and can be infected in humanized mice, allowing pathological effects of HIV variants with different cellular or coreceptor tropism to be systematically compared and studied [33, 34]. HIV infection in these models also leads to CD4 + T cell depletion and has thus been extensively used to study HIV pathogenesis, including how different HIV gene products can affect HIV replication and influence the virus-host interactions that drive HIV disease [35–37]. In models [20] including the severe combined immunodeficient (SCID)-Hu thymus-liver (thy/liv), Hu-HSC (hematopoietic stem cell), and BLT mouse, human immune cells differentiate in vivo from hematopoietic stem cell precursors. This allows hematopoiesis and normal immune cell development to be closely studied, along with the direct or indirect effects of HIV on this process [38]. The types of human cells present in the mice can also be modified by careful selection of human cell/tissue to be transplanted, recipient mouse strain characteristics, and specific humanization procedures, allowing for example the relative contribution of T cells versus macrophages in HIV replication and persistence to be evaluated in an in vivo environment [33, 34].

Early studies of HIV latency in humanized mice [15, 39–41] were performed using a SCID-hu thy/liv model system that largely constrained human cells to the thy/liv implant, which could be used as an ex vivo source of latently-infected cells or as a single-tissue in vivo model. More recent approaches have focused on models such as the BLT mouse, which permits systemic reconstitution with human cells [26], supports the formation of HIV latency following HIV infection [42, 43], and maintain persistent reservoirs of virus including latently-infected CD4 + T cells after treatment with clinically relevant ART regimens [42–45]. In addition to serving as models to explore the establishment of latency and tissue sources of persistent virus during ART, these models also allow testing of efforts to deplete latent virus. For example, agents intended to reverse latency in “kick-and-kill” approaches have been directly evaluated in humanized mouse models [45, 46].

If immune responses against HIV could be sufficiently augmented during ART then virus may be prevented from rebounding if ART is stopped, creating a “functional cure” where the virus is permanently kept in check by the immune system. Alternatively, enhanced anti-HIV immunity may improve the elimination of latent reservoirs if used in conjunction with latency reversing agents in kick and kill approaches. Therefore, various HIV persistence and cure studies are focused on improving the innate and adaptive immune response against HIV. Since the human immune system in modern humanized mouse models contains cytotoxic cell types including CD8 + T cells and some natural killer cells, various efforts to augment anti-HIV immune approaches can also be tested in these systems [14].

Gene therapy approaches targeting human genetic sequences can also be directly evaluated in humanized mice without the need to tailor target sequences to a different model species. The fact that some humanized mouse models support hematopoiesis from CD34 + hematopoietic stem cells through mature immune cells also allows stem cell gene therapy approaches to be evaluated in these models [47]. Furthermore, evaluation of experimental cytokines or other intracellular signaling molecules can be performed without concern that commonly observed [48] inter-species ligand-receptor incompatibilities will occur.

Murine models also typically allow a greater number of animals to be tested with a given experimental approach than non-human primate models, allowing differences in experimental groups to be more easily discerned. Furthermore, small animal sizes allow for easier handling and care, and lower amounts of test drugs or compounds to be used during in vivo studies compared with larger animal studies.

## Limitations to humanized mouse studies

One key limitation to the use of humanized mice is related to their small size. Blood volume and available cell numbers from tissues are lower than is the case with larger animal models or infected humans. Therefore, bleed volumes are typically restricted to a maximum of several hundred microliters per sample depending on animal weight and bleed frequency, with approximately 1-1.5 mL volumes available at necropsy for a 25 g mouse. Humanized BLT mice can harbor millions of resting CD4 + T cells, but if cells resident in individual tissues are assessed, then pooled cells from multiple animals may be required to quantify latently-infected cells in individual tissues.

Another consideration is the xenograft nature of the model in which human immune cells are interacting with mouse tissues. For experiments that involve transplant of mature human immune cells into immunodeficient mice, this often leads to development of graft versus host disease (GVHD) several weeks post-transplant [49]. Models where human cells differentiate from stem cells in the mouse typically do not develop GVHD until much later than this, if at all. The timing of GVHD onset is dependent on experimental specifics, the types of human tissues engrafted, and recipient mouse strain genotype, and these have recently been improved to some extent. For example mice such as the triple knockout (C57Bl/6 with knockout of recombination activating gene 2, common gamma chain, and CD47 genes) have been reported to be healthy for 45 weeks after humanization and could be treated with ART for 18 weeks [50]. Any human-specific differences in tissue architecture or interactions between non-hematopoietic cells and HIV virions or infected cells would also generally not be captured in these models unless additional non-hematopoietic human tissue is implanted [51].

Some changes in the latent or persistent HIV reservoir in humans may take a long time to occur. While a state of HIV “deep latency” has not been defined or characterized at a molecular level, it is possible that the latent proviruses that remain dormant for many years of ART have different characteristics from those that are most abundant early after therapy initiation. Changes to the latent reservoir driven by clonal expansion [8, 52] through homeostasis or antigen-induced proliferation may also not be fully evident over short experimental time periods. The limited lifespan of mice and other constraints of murine model systems including potential GVHD development may restrict their application to these questions that are better addressed with other approaches, for example in larger animal models or using samples from people living with HIV who have undergone long-term ART.

Mice with human immune systems cannot be bred in the same way as genetically modified mice that encode specific human genes. Instead, each one must be transplanted with human cells and/or tissues. For example, commonly used BLT mice can be constructed by implanting fetal thymus and liver tissue under the kidney capsule of immunodeficient mice, followed by irradiation and injection of CD34 + stem cells [26]. This requires microsurgeries conducted by trained investigators and suitable isolation and biohazard containment conditions for maintaining immunodeficient mice which will subsequently be infected with HIV. The introduced stem cells also need to be capable of proliferating and differentiating into a new human immune system. However, the use of fetal tissue sources of stem cells and transplant material, which provide the most robust immune reconstitution in humanized mice, is now subject to substantial restrictions in the United States [53].

Humanized mouse models have also not yet advanced to the point that all aspects of the human immune system are entirely recapitulated. Adaptive immune responses can be elicited but have historically proven relatively weak, and differentiation of stem cells into myeloid and NK cells is limited (reviewed in [20, 54]). Additional advances including, for example, implantation of lung tissue in addition to the human immune system, have recently been described that can improve these adaptive immune responses [51] and further refinements through genetic modification of recipient mouse strains to express human cytokines or other immunomodulatory molecules [48, 54] may lead to improvements in this area.

Finally, while valuable pharmacokinetic and distribution studies of anti-HIV drugs can be conducted in humanized mice [55], the basal metabolic rate of mice and humans differs, along with drug pharmacokinetic and pharmacodynamic properties [56] which must be considered when designing experiments.

## Conclusions

The application of humanized mouse models to studies of HIV persistence and latency offers a complementary approach to clinical studies in people living with HIV and strategies using non-human primates. These models have provided insights into many aspects of HIV biology, and their utility as basic science and translational tools in HIV persistence and cure research is substantial. However, as is always the case in experimental biology, understanding the caveats of different model systems and selecting the best approach to address the scientific question under investigation is critical. As the saying goes: “Always pick the right tool for the job at hand”.

## Data Availability

Not applicable.

## Abbreviations

ART: Antiretroviral therapy
HIV: Human Immunodeficiency Virus
SIV: Simian Immunodeficiency Virus
SHIV: Simian-Human Immunodeficiency Virus
BLT: Bone-marrow liver thymus
HSC: Hematopoietic stem cell
GVHD: Graft versus host disease
